# Endoscopic third ventriculostomy in children with a fiber optic neuroendoscopy

**DOI:** 10.1007/s00381-017-3679-4

**Published:** 2017-12-16

**Authors:** Wenjun Shen, Hasan R. Syed, Gurpreet Gandhoke, Roxanna Garcia, Tatiana Pundy, Tadanori Tomita

**Affiliations:** 1 Division of Pediatric Neurosurgery, Ann & Robert H. Lurie Children’s Hospital of Chicago, 225 E. Chicago Avenue, Chicago, IL 60611-2605 USA; 2 Division of Pediatric Neurosurgery, Children’s hospital of Fudan University, Shanghai, China

**Keywords:** Endoscopic third ventriculostomy, Hydrocephalus, Children, Surgical technique

## Abstract

**Objective:**

Endoscopic third ventriculostomy (ETV) provides a shunt-free treatment for obstructive hydrocephalus children. With rapidly evolving technology, the semi-rigid fiber optic neuroendoscopy shows a potential application in ETV by blunt fenestration. A retrospective analysis of our experience is reviewed.

**Methods:**

The authors review infants and children who underwent ETV using this technique from June 2004 to June 2016 with radiological and clinical follow-up done by a single surgeon. Patients who underwent ETV with channel scope were excluded. Demographic variables and operative reports were collected. Improvement of preoperative symptoms and avoidance of additional cerebrospinal fluid (CSF) diversion procedures were considered a success. The ETV success score (ETVSS) was used to correlate with clinical outcomes.

**Results:**

A total of 79 patients were included with a mean age of 8.3 ± 5.5 years, and 40.5% were female. The mean clinical and radiographic follow-up was 38.6 ± 40.9 months. The overall complication rate was 6.3%, while 73.4% were considered successful. The ETV failure cases received conversion to ventriculoperitoneal shunt or redo of ETV with a median time of 2 months. The mean ETV success score was 74.3 ± 11.8 with positive correlation between success rate (*P* < 0.05). Kaplan-Meier failure-free survival rates of 30-day, 90-day, 6-month, 1-year, and 2-year were 89.9, 83.5, 78.5, 75.9, and 74.6%. Eight patients required redo ETV, and five of these patients required eventual shunt placements. Approximately 61.9% of failure occurred within 3 months. Patients with post-intraventricular hemorrhage (IVH) /infection, and age younger than 12 months had the poorest outcome (*P* < 0.05).

**Conclusions:**

Blunt dissection of the third ventricle floor under endoscopic vision with the stylet tip of a fiber optic neuroendoscopy is safe and requires less equipment in the pediatric population. This technique is successful with an optimistic long-term outcome except for infants and the post-IVH and infectious subgroups.

## Introduction

Endoscopic third ventriculostomy (ETV) is the most commonly performed endoscopic procedure in neurosurgery for obstructive hydrocephalus [1]. It provides a more physiological restoration of cerebrospinal fluid (CSF) between ventricular cavities and the interpeduncular cistern. A prospective, multicenter study published in 2016 revealed the success rates of ETV of 6 months and 2 years are 66.7 and 57.8%, respectively [2]. More and more centers around the world have used ETV to treat hydrocephalus. Kulkarni et al. first described the ETV success score (ETVSS) in 2009 [3], and this statistical model has shown validity and calibration in predicting ETV success within the first 6 months [2].

Although there is no consensus on the instrumentation and the technique to be used, the core is creating and widening the hole on the floor of the third ventricle. Rigid lens scopes with working channel provide the advantage of better visualization of anatomical structures, but are burdened by larger instrument size at the time of ventriculostomy. These rigid lens scopes with working channel are placed into the ventricle through a peel away catheter or trocar of 12.5 Fr. to 19 Fr. (4.1 to 6.0 mm) in diameter. Several methods have been used to perform fenestration and its widening at the floor of the third ventricle, including mainly the alligator clamp, Fogarty balloon, laser, or water jet dissection [4, 5]. In all of the abovementioned techniques, a working channel is required to allow for the use of the instrumentation. These techniques need larger burr hole for instrumentation, and leave larger needle tract. Although the use of instrumentation under endoscopic guidance claims to be safer because of the direct visualization of the instruments, vascular damage can nonetheless occur [2, 6].

In order to minimize the burr hole and needle tract, we have used regular sized ventricular catheter as a sheet for semi-rigid fiber optic neuroendoscopy for ETV. The semi-rigid fiber optic scope has 1.14 mm in diameter, and it is inserted into a ventriculostomy catheter of 2.3 to 2.8 mm in diameter. We present our results with the flexible neuroendoscope and blunt dissection technique for ETV in 85 patients.

## Methods

### Patient selection and data source

The authors conducted a retrospective analysis of infants and children who underwent ETV at Ann & Robert H. Lurie Children’s Hospital of Chicago from June 2004 to June 2016, which were performed with a uniform technique by a single surgeon (corresponding author). Hydrocephalus was untreated previously in all except for three patients with existing VP shunt. Among latter, ETV was performed at the VP shunt malfunction in two with a shunt removal and another without shunt revision. Demographic variables included gender, age at ETV, etiology of hydrocephalus, and prior shunt history. Operative reports were collected and the ETVSS were calculated. Patients were followed clinically and radiologically. All patients had one radiological study (CT or MR) in the early post-operative period and 1 month after the ETV. The subsequent follow-up was scheduled according to the clinical response and to the course of the etiology of the hydrocephalus. Success of ETV was determined on the basis of the last follow-up: the improvement of preoperative symptoms with or without reduction of ventricular size on radiological examination was considered a success, while the need for insertion of a VP shunt/redo of ETV or radiographic progression of hydrocephalus were considered a failure.

### Surgical technique

**Fig. 1:**
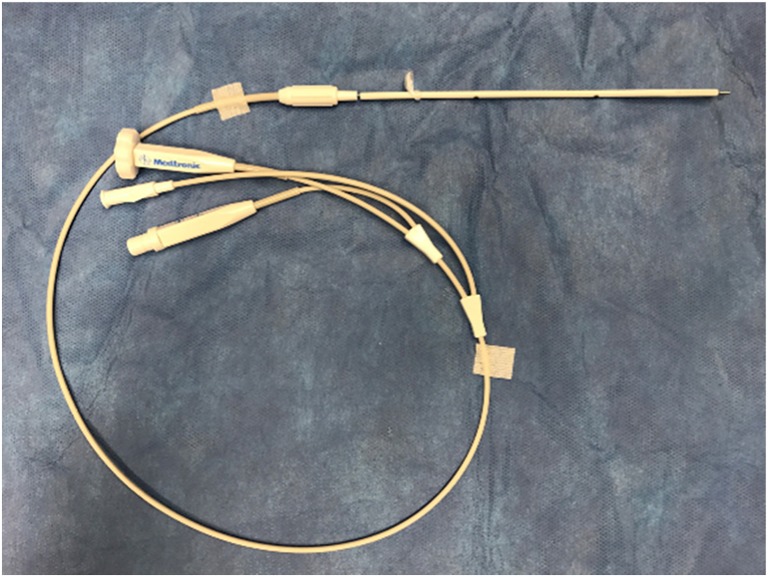
NeuroPen fiber optic neuroendoscopy inserted into a ventricular slotted innervision catheter

A sterile 15-cm semi-rigid (1.14 mm in diameter) stylet neuroendoscope (Neuropen, Medtronic PS Medical, Goleta, CA, USA) is inserted into a ventricular slotted innervision catheter (2.8 mm in outer diameter) with a distal slit tip (Innervision Ventricular Catheter, Medtronic PS Medical, Goleta, CA, USA) (Fig. 1). All procedures were performed with the patient intubated, under general anesthesia, and in the supine position. A right frontal burr hole is placed at or just anterior to the coronal suture and 2–3 cm lateral to the sagittal midline usually on the right side.

After opening the dura, the neuroendoscope and ventricular catheter system are introduced into the frontal horn of the lateral ventricle using a free-hand technique or with the guidance of the neuro-navigation. Standard anatomical landmarks within the lateral ventricle are identified and the third ventricle is accessed through the ipsilateral foramen of Monro.

**Fig. 2:**
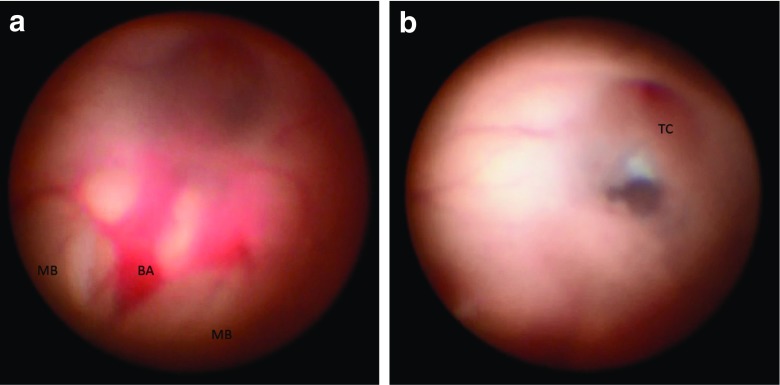
**a**, **b** Surgical views of the floor of the third ventricle through a NeuroPen endoscope in a 4-year-old girl with diffuse intrinsic pontine glioma and obstructive hydrocephalus, before (**a**) and after (**b**) endoscopic third ventriculostomy (ETV). The basilar artery (BA) and hypertrophic belly of the pons are visible between the mammillary bodies (MB) through semitransparent floor (**a**). A stoma after ETV is shown behind the vascular stain of the tuber cinereum (TC) (**b**). Note a rapid thickening of the floor of the third ventricle after ETV

The floor of the third ventricle is inspected, the basilar artery may be identified by its transmitted pulsation, though sometimes it can be visualized through a thin floor. A stoma is placed in the center of the floor posteriorly to the vascular stain of the tuber cinereum and anteriorly to the mammillary bodies. The ependymal layer is first scored by the tip of the endoscope, and then identified the location by withdrawing the scope tip slightly. Once the scored site is satisfactory, the endoscope tip is advanced to perforate the pia. The careful advancement of the neuroendoscope allows us to visualize the progressive thinning of the floor and the eventual appearance of the pia. During the tip advancement, if reddish structures appear, further perforation is stopped, because it is likely heading to the basilar artery. During this time, the structures of the interpeduncular and prepontine cistern became visible through the transparent pia layer. Once the endoscope tip is in the cistern, the stoma is widened mechanically, by advancing the catheter over the endoscope further deeper into the prepontine space, and sliding the catheter sideways over the clivus. In the subarachnoid space, the Liliequist’s membrane is fenestrated in a similar fashion. The prepontine space is carefully inspected in order to break any arachnoidal adhesions mechanically. After withdrawing the scope and the catheter into the third ventricle, the stoma is easily visualized (Fig. 2). Intraoperatively, the third ventriculostomy is judged successful if the edges of the stoma flap freely with the passage of CSF, and the basilar artery, its branches, and the belly of the pons are visualized without any overlying membrane. Though there is no validated way to measure the size of the stoma, a good intraoperative reference is the diameter of the basilar artery or half a distance between the tuber cinereum and the anterior mammillary line, which are considered to be sufficient size of the stoma.

The ventricular catheter is routinely left in the lateral ventricle and connected to the Ommaya reservoir in the subgaleal space postoperatively.

### Statistical analysis

Continuous variables were expressed as the mean ± standard errors of the mean (SEM) and categorical variables are shown as raw values with percentages. Failure-free survival and curves were estimated using the Kaplan-Meier method and compared with the log-rank test (Mantel-Cox). *P* values < 0.05 were considered statistically significant. All analyses were performed using the SPSS Version 19.0 software.

## Results

A total of 85 ETV cases’ meet inclusion criteria but 6 patients were lost to follow-up due to inadequate postoperative information. The characteristics of the 79 ETV follow-up cases are listed in Table 1. The mean age is 8.3 ± 5.5 years old (2 month to 20 years old), and the male-to-female ratio was 1.5. The predominant etiology for hydrocephalus was tumor (47 cases, 59.5%) (Table 2), followed by aqueductal stenosis (10 cases, 12.9%), unbiopsied tectal lesion (9 cases, 11.4%), post-intraventricular hemorrhage (IVH) (3 cases, 3.8%), myelomeningocele (2 cases, 2.5%), post-infection (1 case, 1.3%), and other causes in the remaining 7 cases (8.9%).

**Table 1:** Summary of characteristics of patients

Variable	No. of cases (%)
Previous shunt	3 (3.8%)
Age at ETV	
< 1 month	0 (0.0%)
1 to < 6 months	4 (5.1%)
6 to < 12 months	7 (8.9%)
1 to < 10 years	33 (41.8%)
≥ 10 years	35 (44.3%)
Etiology	
Tumor	47 (59.5%)
Aqueductal stenosis	10 (12.7%)
Tectal lesion	9 (11.4%)
Post-IVH	3 (3.8%)
Myelomeningocele	2 (2.5%)
Post-infection	1 (1.3%)
Other	7 (8.9%)
ETVSS	
10	0 (0.0%)
20	1 (1.3%)
30	0 (0.0%)
40	1 (1.3%)
50	2 (2.5%)
60	6 (7.6%)
70	29 (36.7%)
80	28 (35.4%)
90	12 (15.2%)

**Table 2:** Tumor etiology of hydrocephalus

Anatomy region	Etiology	*N* = 47
Pineal region		*N* = 8
	Pineoblastoma	4
	Germinoma	2
	AT/RT	1
	Teratoma	1
Cerebellar + 4th ventricle + CPA		*N* = 16
	Medulloblastoma	8
	Ependymoma	4
	LGG	2
	Diffuse hystiocytosis	1
	Lymphoma	1
Mid brain + brainstem + thalamus		*N* = 22
	LGG	10
	HGG	5
	DIPG	4
	NF	2
	PNET	1
Suprasellar cistern		*N* = 1
	LGG	1

*AT/RT* atypical teratoid/rhabdoid tumor, *CPA* cerebellopontine angle, *LGG* low-grade giloma, *HGG* high-grade giloma, *DIPG* diffuse intrinsic pontine glioma, *NF* neurofibroma, *PNET* primitive neuroectodermal tumors

Eight patients required redo ETV, three redos in 1 child, and a single redo in 7 children, thus accounting for a total of 95 procedures among 85 patients. The overall complication rate was 6.3% (6 out of 95 procedures): 1 intraoperative venous hemorrhages required a temporary external ventricular drainage (EVD), 2 intraoperative minor hemorrhages without EVD, and 3 complicated by postoperative staph ventriculitis. Five out of six patients with complications required the placement of a VP shunt eventually.

**Fig. 3:**
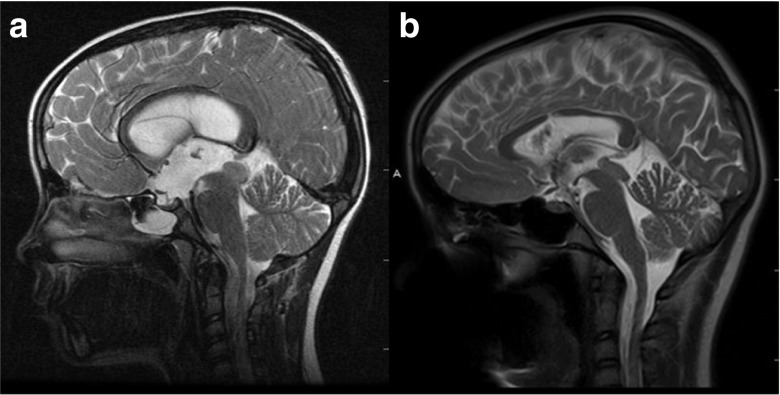
An 8-year-old female presented with headaches and papilledema. Preoperative T2-weighted MR (**a**) shows a tectal plate lesion and obstructive hydrocephalus. T2-weighted MR (**b**), 7 years after ETV, shows a reduction of the ventricles with flow void at the floor of the third ventricle

The mean follow-up time is 38.6 ± 40.9 months (range 1–149 months), with approximately 73.4% technique success. Twenty-one cases (26.6%) had ETV failure and were converted to VP shunt or had ETV redo with a median time of 2 months (range 1–48 months). The causes of failure were explored when redo or VP shunt with an endoscopy in 19 patients (summarized in Table 3). Most of the failure cases (13 cases, 61.9%) occurred in the early post-operative period within 3 months. One patient had acute neurological deterioration with bradycardia 19 months after the ETV, which we considered late ETV failure. The mean age among ETV failure cases was significantly younger than shunt-free cases (5.5 ± 5.3 vs 9.4 ± 5.2, *P* < 0.01). Patients with ETV due to post-IVH or infectious hydrocephalus had poorer outcome, only 25% success rate, and children younger than 12 months only have a 27.3% technique success rate (Table 4). Only one of three patients with existing shunt failed after ETV. The success rate for tumor, tectal lesion, and aqueductal stenosis is 80.4, 77.8, and 70.0%, respectively (Table 4). Follow up MR show various degree of reduction in the ventricle size, but none showed slit-like ventricle (Fig. 3).

**Table 3:** ETV failure reasons

Reasons	*N*
Stoma closure	7
Communicating hydrocephalus	4
Lilliquist membrane	2
Tumor progression	2
Subdural CSF collection	2
Infection	1
Ventricle abnormality	1
N/A	2

**Table 4:** Incidence of success/failure according to etiology and age

Diagnosis	All patients	Success	Failure	*p*
Tumor	56	45 (80.4%)	11 (19.6%)	0.029
Tectal lesion	9	7 (77.8%)	2 (22.2%)	0.753
Aqueductal stenosis	10	7 (70.0%)	3 (30.0%)	0.793
Post-IVH/infection	4	1 (25.0%)	3 (75.0%)	0.024
Infant (≤ 12 months)	11	3 (27.3%)	8 (72.7%)	< 0.001

**Fig. 4:**
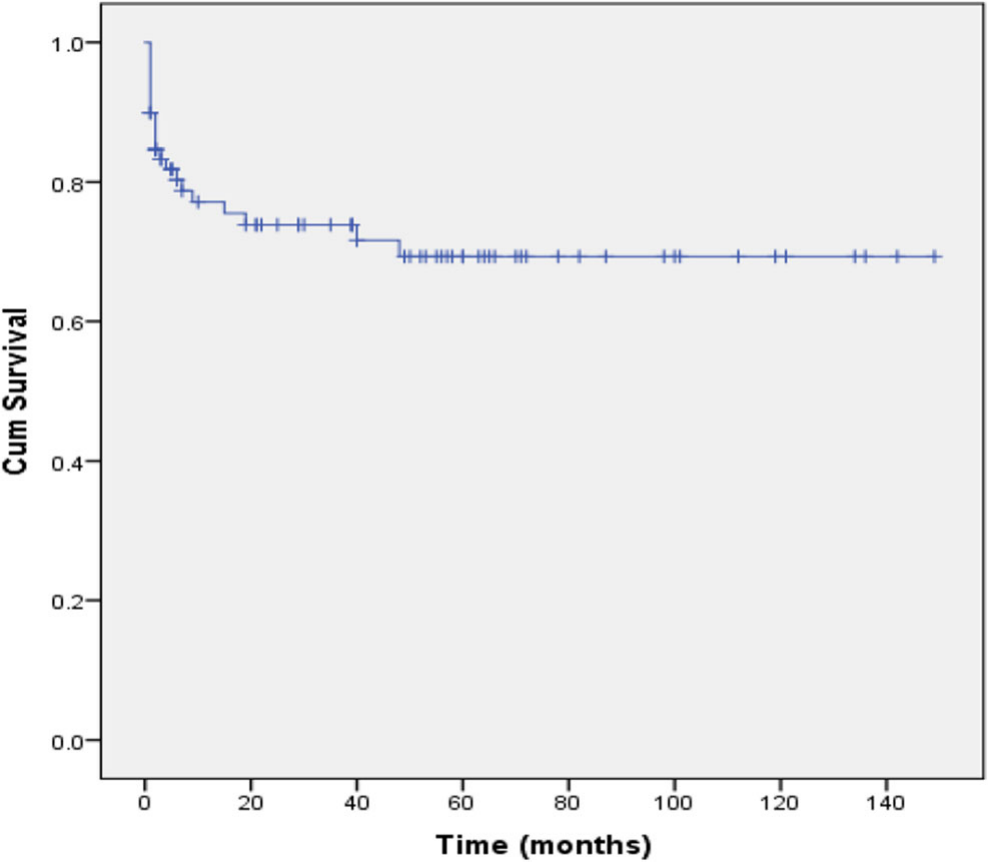
ETV failure-free survival curve

The mean ETVSS in all patients was 74.3 ± 11.8 (Table 5). There was a correlation between high ETVSS and technique success (*P* < 0.05). The area under receiver operator characteristic curve for ETVSS as an independent predictor of successful outcome was 0.72 (95% CI 0.57–0.87, sensitivity 98.3%, specificity 17.7%). Kaplan-Meier failure-free survival rates of 30-day, 90-day, 6-month, 1-year, and 2-year were 89.9, 83.5, 78.5, 75.9, and 74.6%, respectively. Figure 4 shows the Kaplan-Meier survival curve for time to ETV failure.

**Table 5:** Relationship between ETVSS and failure-free survival

ETVSS	*N*	Failure-free survival (%)
≤ 40	2 (2.6%)	0
50–70	37 (46.8%)	70.3
≥ 80	40 (50.6%)	80.0

## Discussion

To access the ventricular system endoscopically, normal brain parenchyma needs to be traversed. Minimizing this collateral damage is the main theme behind using this minimally invasive technique. Although the rigid lens scopes provide high-quality images and angled-variety, the advances in the field of fiber optics have led to significant improvements in image resolution. They can be inserted into the lumen of ventricular catheter without trocar or peelaway catheter which needs a larger burr hole and needle tract. The use of ventricular catheter as a sheet for ventricoscope is particularly useful when the ventricles are small or at the time of shunt revision. The endoscopic procedure is easily performed by a single surgeon without using any endoscope holder or requiring to tackle various other simultaneous instruments. Being semi-rigid, the fiber scope gives the surgeon the opportunity to bend in small angles and adjust to respective target trajectory with precision under the tip of the scope if necessary.

Moreover, when a patent stoma is achieved and widened by means of perforating instruments like forceps, the tip of the Fogarty catheter, or electrocautery, a working channel is essential. Injury to perforating vessels has been reported in such ways [6]. At the time of using the fiber neuroendoscope during the fenestration of the floor, one should be familiar with the anatomy of the floor of the third ventricle and avoid any reddish structure during the advancement of the tip of the endoscope during the perforation of the floor. The safest way is initially scoring the ependymal layer of the floor of the third ventricle by the tip of the endoscope to identify the location and direction of intended ETV.

The diameter of the scope has been linked to post-operative subdural collections in young children [7], and is also crucial to reduce the risk of damage to neural structures within the ventricles, such as the fornix, particularly in the presence of distorted anatomy like a scarred foramen of Monro [8] encountered in cases of children with history of prior shunt. It has been described that ETV has a 5~19% complication rate containing venous hemorrhage, basilar artery perforation, subarachnoid or intraventricular hemorrhage, thalamic or hypothalamic contusion, fornix or midbrain injury, memory deficit, diabetes insipidus, weight gain, precocious puberty, bradycardia, hemiparesis, 3rd nerve palsy, meningitis, ventriculitis, superficial infection, CSF leak, subdural CSF collection, altered consciousness, and herniation syndrome [9–11]. We have a 6.3% complication rate without any injury to the endocrinological, neural, or vascular structures. Limiting and reducing complications are essential, as the incidence of failure of ETV is related to early complications as noted in our previous experience [12]. The present analysis validates this assumption, because all procedures complicated by intraoperative hemorrhage or early post-operative infection ultimately lead to the placement of a VP shunt (*P* < 0.05). Our cohort has a similar mean ETVSS when compared to multicenter data [2] (74.3 vs 74.8) with no bias in the candidates selection. The comparison of 2-year failure-free survival rates between our series and multicenter data, 74.6 vs 57.8% respectively (*P* < 0.05), indicates certain advantages with fiber optic neuroendoscopy blunt dissection.

Although there is no way to measure the size of the stoma intraoperatively, the size of stoma is expected to correlate with the success of ETV: Kombogiorgas and Sgouros measured the relative size of stoma as the percentage of stoma diameter to the distance percentage of stoma between posterior clinoid and basilar artery. They reported that stoma size may correlate with success without statistical significance [13]. We achieve an improved stoma size by advancing the catheter over the endoscope and then sliding neuroendoscope sideways between the basilar artery and the clivus. Thick ventricular floor or arachnoid adhesion were considered one of the negative intraoperative findings for poor ETV outcome [14]. This technique allows to easy conversion to shunt in the event that intraoperative findings do not favor an ETV. We excluded four such intention failure cases from this study who had placement of VP shunt after aborting ETV. These patients represent 4.5% out of 89 ETV intentions. Promising data has been shown by the use of the FIESTA sequence of the MR in the preoperative assessment of the subarachnoid space, which will be useful in selecting favorable patients for ETV [15]. There is little information in the literature about ETV redo; while one paper from Germany in adult reported an 87.5% redo success rate [16], we have a 37.5% redo successful rate from our cohort of eight patients.

The success rate of this minimally invasive procedure was 73.4% in our series and is comparable to the other studies [2, 17]. Several factors have been shown to affect the outcome of ETV, in particular the age of patient at the time of operation, the cause of hydrocephalus, and prior shunting, which are factors that are used to calculate the ETVSS [2, 3]. Our previous paper on ETV has confirmed relative lower success rate in younger children [18]. We found that IVH and infection were related to a negative outcome statistically, which is similar with the literature [2]. Radiological and intraoperative factors predictive of failure have also been reported [14, 19–21]. The pulsation of the floor is considered critical because it provides an indirect measure of the subarachnoid space, which indicates brisk CSF flow from the ventricle to the prepontine space [13]. Visualization of a naked basilar artery is a positive predictor of ETV success [2]. We were able to clearly identify the basilar artery in every case, only two failure cases had remaining membrane confirmed at the redo ETV (Table 3). The present experience shows that triventricular hydrocephalus secondary to tumor obstruction has more benefit from ETV (Table 4). The aqueductal stenosis cases had a 70.0% success rate, while it is comparable to above 60% reported in the literatures [22, 23]. Interestingly, in neoplastic cases, the success rate increased to 80.4% (*P* < 0.05). Although 15 tumor progression cases had shorter follow-up time (14.2 ± 16.8 months), patients with neoplastic cases as a whole had similar follow-up time compared to other etiology. After excluding this bias, our result confirms that obstructive hydrocephalus secondary to tumors either in pineal or posterior fossa regions is a good indication for ETV, as confirmed with the other reports [24–26]. On the contrary, if the tumor occupied the floor of the third ventricle or the prepontine cistern [27], we found a 33.3% (one out of three) success rate in this subgroup. While confounded by a small sample size, we believe pathology in this region is a contraindication to ETV.

The majority of ETV failure occurred in early post-operative period [2, 28]; 13 out of 21 (61.9%) within the first 3 months and 17 out of 21 (81%) within the first 6 months in our series. The cause of late rapid deterioration of ETV has not been fully studied, and it usually happened 2.5 years on average after initial ETV [29]. A population-based analysis showed that a second round of failure occurred around 3 years after the initial ETV procedure in children between 1 and 10 years of age with tumor or aqueductal stenosis [30]. We had one late rapid ETV failure at 19th month, but a second failure peak could not be found in our cohort. A total of 11 cases died from tumor progression in the follow-up period (mean follow-up time 14.2 ± 16.8 months), which is statistically shorter than the other subgroups (*P* < 0.05). The poor survival prognosis in this subgroup may deprive the chance to reflect the second failure round.

In this series, nearly every patient had an Ommaya reservoir connecting to ventricular catheter following ETV, which allows us to gain access to the intracranial CSF, to monitor ICP and to drain the CSF if necessary. Moreover, potentially catastrophic acute hydrocephalus after ventriculostomy closure can be managed with a ventricular access device in place [29, 31, 32]. Radiological evidence of patent stoma could be achieved by injection contrast through the reservoir. The most failure reason is stoma closure due to the scaring, counting 33.3% (7 out of 21) of all the failure cases (Table 3), which is similar to a 42.8% rate reported in the literature [16]. The other failure reasons were combined communicating hydrocephalus, Liliequist membrane, tumor progression, subdural CSF collection, infection, and ventricle abnormality (Table 3).

The size of the stoma created at the floor of the third ventricle may affect the ETV survival. The larger the soma, the lower the failure rate would be [2]. The normal aqueduct of Sylvius is quite narrow. The ranges of luminal area of cerebral aqueduct in children was reported from 0.15 mm^2^ with a mean size of 0.5 mm^2^, while its length ranges from 12.8 mm at birth to 18 mm after 8 years old [33]. We have used a slotted innervision catheter rather than regular catheter as it provided a larger diameter [12]. Slotted innervision catheter with an outer diameter of 2.8 mm produces a surface area of a hole of 6.15 mm^2^ by a single puncture, whereas regular innervision catheter was 2.5 mm and 4.9 mm^2^, respectively. So our technique has provided adequate patency mechanically, much larger than the physiological size of the aqueduct. However, the difference between the normal cerebral aqueduct and the ETV stoma is that the former is lined by normal ependymal and the later has glial tissue and leptomeninges in the surrounding tissue. Therefore, over time the ETV can be closed due to the development of meningocerebral cicatrix, while the intact ependymal layer does not adhere under normal condition, which attributes to our one-third redo cases.

Studies have revealed that the ETV success rate is highly dependent on age [2, 3, 18], we also found a statistic younger age in all the failure cases. The recent prospective comparison from the International Infant Hydrocephalus Study showed that infants (< 24 months old) with aqueductal stenosis would benefit more likely from a shunt than ETV [34]; the actual success rates for ETV versus shunt at 3, 6, and 12 months were as follows: 68 vs. 95%, 66 vs. 88%, and 66 vs. 83%. The infants in our cohort had a 27.3% chance of ETV success, while the risk of failure increased for ages younger than 6 months with a 25% chance, both consistent with the other reports [34, 35]. As the initial outcome of treating young infant with ETV/choroid plexus coagulation (CPC) is promising [36], some recommend ETV/CPC as the first line of treatment for infants’ hydrocephalus [37].

## Conclusions

Blunt dissection of the third ventricle floor under endoscopic vision with the stylet tip of a fiber optic neuroendoscopy to perform ETV is safe, cost-effective, and requires less equipment in experienced hands. Inability of execution of ETV was 4.5% among attempted. This technique is successful with an optimistic long-term outcome but may be challenging in certain populations, including infants and the post-IVH and infectious subgroups.
